# Self-rated health of primary care house officers and its relationship to psychological and spiritual well-being

**DOI:** 10.1186/1472-6920-7-9

**Published:** 2007-05-02

**Authors:** Michael S Yi, Joseph M Mrus, Caroline V Mueller, Sara E Luckhaupt, Amy H Peterman, Christina M Puchalski, Joel Tsevat

**Affiliations:** 1 Section of Outcomes Research, Division of General Internal Medicine, Department of Internal Medicine, University of Cincinnati Medical Center, Cincinnati, OH, USA; 2 Institute for the Study of Health, University of Cincinnati Medical Center, Cincinnati, OH, USA; 3 Cincinnati Children's Hospital Medical Center, Cincinnati, OH, USA; 4 HSR&D Service, Cincinnati Veterans Affairs Medical Center, Cincinnati, OH, USA; 5 Department of Psychology, University of North Carolina at Charlotte, Charlotte, NC, USA; 6 Department of Medicine, George Washington University Medical Center, Washington, DC, USA; 7 George Washington Institute for Spirituality and Health, Washington, DC, USA; 8 Tibotec Therapeutics Clinical Affairs, East Bridgewater, NJ, USA

## Abstract

**Background:**

The stress associated with residency training may place house officers at risk for poorer health. We sought to determine the level of self-reported health among resident physicians and to ascertain factors that are associated with their reported health.

**Methods:**

A questionnaire was administered to house officers in 4 residency programs at a large Midwestern medical center. Self-rated health was determined by using a health rating scale (ranging from 0 = death to 100 = perfect health) and a Likert scale (ranging from "poor" health to "excellent" health). Independent variables included demographics, residency program type, post-graduate year level, current rotation, depressive symptoms, religious affiliation, religiosity, religious coping, and spirituality.

**Results:**

We collected data from 227 subjects (92% response rate). The overall mean (SD) health rating score was 87 (10; range, 40–100), with only 4 (2%) subjects reporting a score of 100; on the Likert scale, only 88 (39%) reported excellent health. Lower health rating scores were significantly associated (P < 0.05) with internal medicine residency program, post-graduate year level, depressive symptoms, and poorer spiritual well-being. In multivariable analyses, lower health rating scores were associated with internal medicine residency program, depressive symptoms, and poorer spiritual well-being.

**Conclusion:**

Residents' self-rated health was poorer than might be expected in a cohort of relatively young physicians and was related to program type, depressive symptoms, and spiritual well-being. Future studies should examine whether treating depressive symptoms and attending to spiritual needs can improve the overall health and well-being of primary care house officers.

## Background

Although physicians generally undergo residency training when they are in their physical prime, residency is physically and emotionally demanding. [1-17] A number of cross-sectional and longitudinal studies over the past decades have examined the negative impact of residency training on fatigue level, [1,14,16,17] stress, [1-13,15] and even physiologic measures (e.g., blood pressure and heart rate) [2] in resident physicians. In a recent review, levels of burnout among house officers were found to be high, [18] with the potential to adversely impact patient care. Residency training may also adversely impact the self-health care practices (e.g., consuming too much alcohol or working through acute illness) [19,20] and self-perceived health of house officers. [19-21] Previous investigations have reported that the health risk during training may vary based on certain demographic factors [3,13] and residency-related characteristics (e.g., program type and post-graduate year). [3,9,10,12,13]

Certain other personal characteristics, such as religiosity and spirituality, have been related to psychological and physical health. [22,23] Although there continues to be ongoing discussions over their respective definitions, the terms "religion" and "religiosity" have been used more recently to describe formal and institutional expressions of faith [22,24] whereas "spirituality" has been used to describe an individual's sense of coherence and connectedness to the truth or the divine. [22,24] The salutary effects of spirituality/religion on physical health have been proposed to work through a number of mechanisms, including beneficial physiologic mechanisms (e.g., lower blood pressure, lower cholesterol level) [25] and health promoting behaviors. [25] Spirituality and religion may also strengthen coping mechanisms against the negative long-term effects of stress by establishing a sense of meaning in life [23] and increasing involvement in social networks. [23] A previous study showed potentially beneficial health effects of certain religious characteristics on young doctors' long-term health outcomes. [26] However, the effects of spirituality/religion on health may not always be salutary. [25,27] Spirituality/religion, then, may be one factor, along with other psychosocial and residency-related factors, related to the self-rated health of house officers. [28-34] We therefore investigated the self-rated health of a contemporaneous group of house staff in 4 different primary care residency programs, and examined factors related to their health. We hypothesized that distinct dimensions of religion, religiosity, and spirituality may have salutary effects on residents' overall self-rated health, [25,26] and thus may serve as potential targets for intervention.

Our specific objectives were: 1) to assess how primary care house officers rate their own health; and 2) to assess how self-rated health is related to demographic and residency characteristics, mood, and specific dimensions of spirituality and religiosity.

## Methods

### Study Participants

Between July 2003 and November 2003, we recruited house officers from 4 primary care residency-training programs [35] at the University of Cincinnati and Cincinnati Children's Hospital Medical Centers in Cincinnati, OH: internal medicine, pediatrics, combined internal medicine-pediatrics, and family medicine. We administered a questionnaire during each program's annual In-Training Exam; all participating house officers were excused from overnight call duties on the night prior to the exam. The respondents' identities were kept completely anonymous and participation was voluntary. For completing the survey, house officers received a free lunch and a $5 gift certificate to a local coffee shop. The institutional review boards at the University of Cincinnati Medical Center and Cincinnati Children's Hospital Medical Center approved the study.

### Instruments

#### Outcome Measure

We assessed house officers' current self-reported overall health by using a rating scale with a range of 0 (labeled "death") to 100 (labeled "perfect health"). [36,37] We also asked a single question about their general health: "In general, would you say that your health has been Excellent, Very Good, Good, Fair, or Poor." [36]

#### Independent Measures

##### Residents' Demographic and Residency Characteristics

We assessed house officers' demographic and residency-related characteristics. Specifically, we asked questions regarding age, sex, race/ethnicity, post-graduate year level, program type (pediatrics, internal medicine, combined internal medicine-pediatrics, or family medicine), and current rotation (inpatient ward/intensive care unit versus non-inpatient-based rotation). We assumed that all house staff physicians had similar education levels and incomes, so we did not ask about education and income.

##### Residents' Mood

Depressive symptoms were measured by using the 10-item Center for Epidemiologic Studies-Depression Scale (CESD-10). [38,39] The CESD-10 has been used extensively in a number of general and chronically ill populations and asks the subject to respond to 10 statements as they have pertained to themselves during the past week. Scores on each item are summed (range 0–30), with higher scores representing greater levels of depressive symptoms.

##### Residents' Religious Affiliation, Religiosity, and Spirituality

We first asked a question modeled after the religious preference question used in Gallup surveys, "What is your religious affiliation?" [40] We then administered the 5-item Duke Religion Index [41] to measure 3 dimensions of religious activity: organized religious activity (scored from 1–6, with higher scores indicating more frequent activity), non-organized religious activity (scored from 1–6, with higher scores indicating more frequent activity), and intrinsic religiosity (subjective views on religion and religious experience; scored from 3–15, with higher scores indicating greater levels of intrinsic religiosity). We used an adapted 10-item version of the Brief RCOPE [42] (modified with the aid of K. Pargament, PhD, the developer of the instrument) to assess the positive and negative roles of religion in coping with the stresses of residency training (in lieu of coping with chronic illness). Positive religious coping mechanisms (e.g., collaborative religious coping, benevolent religious reappraisals) are scored from 6–24, with higher scores indicating greater use of positive religious coping mechanisms, and negative religious coping mechanisms (e.g., feelings of spiritual abandonment) are scored from 4–16, with higher scores indicating more frequent use of negative religious coping mechanisms. We also used 3 subscales from the full RCOPE to assess interpersonal religious discontent (scored from 3–12, with higher scores indicating greater discontent with clergy/religious institutions), religious support seeking (scored from 5–20, with higher scores indicating greater levels of seeking support from clergy or members of the congregation), and spiritual support seeking (scored from 4–16, with higher scores indicating greater levels of seeking support from or connecting with a higher power). We measured spiritual well-being by using the 23-item Functional Assessment of Chronic Illness Therapy-Spiritual Well-being-Expanded (FACIT-SpEx) scale, [43] which assesses faith (comfort and strength in one's beliefs) and meaning/peace (sense of meaning, purpose, and peacefulness in life). The FACIT-SpEx is scored from 0–92, with higher scores indicating greater overall spiritual well-being.

### Statistical Analysis

Descriptive statistics included means, standard deviations, medians, and 25^th ^and 75^th ^percentiles. Due to relatively small numbers of subjects in specific minority groups, we dichotomized race as Caucasian versus non-Caucasian. We categorized religious affiliations into Christian, Jewish, Other, and None/Undesignated for our analyses. Univariate analyses included χ^2 ^tests to compare proportions and Spearman's correlation coefficients, Student's t-tests, or Wilcoxon rank-sum tests, as appropriate, for continuous variables.

We conducted a multivariable analysis in which independent variables associated with health rating scores at a P < 0.20 level in univariate analyses were entered into a backward elimination linear regression model. Variables associated with health ratings at a P < 0.05 level in the backward elimination were retained in the final model. Due to the relatively skewed nature of the health rating scores, we examined our final multivariable model using log transformed health rating scores. We found no significant change in the relationship between the outcome and independent variables, so we report the non-transformed results. Regression diagnostics were examined for multicollinearity and no problems with multicollinearity were found. All analyses were performed by using SAS software, version 8.2 (SAS Institute, Inc., Cary, NC).

## Results

Of a total of 247 house officers in the 4 primary care training programs, 227 subjects completed the questionnaires (92% response rate). Their mean (SD) age was 28.7 (3.8) years; 131 (58%) were female; 167 (74%) were white; 165 (73%) were Christian; and 112 (49%) were pediatric residents, 62 (27%) were internal medicine residents, 26 (12%) were combined internal medicine-pediatric residents, and 27 (12%) were family medicine residents (Table 1).

**Table 1:** Residents' Characteristics

**Variable**	**N (%)**	Mean Health Rating Scale Score (SD)*	**P-value**
Sex			NS
Male	95 (42)	87 (9)	
Female	131 (58)	86 (10)	
Race/ethnicity			NS
Caucasian	167 (75)	87 (9)	
Minority	58 (25)	85 (12)	
Post-Graduate Year (PGY)			0.03
PGY-1	70 (31)	87 (10)	
PGY-2	70 (31)	84 (11)	
PGY-3	72 (32)	89 (9)	
PGY-4 and greater	14 (6)	84 (8)	
Residency Program Type			< 0.001
Internal Medicine	62 (27)	81 (14)	
Pediatrics	112 (49)	88 (7)	
Internal Medicine-Pediatrics	26 (12)	89 (6)	
Family Medicine	27 (12)	88 (9)	
Clinical Rotation			NS
Inpatient	145 (65)	86 (10)	
Non-inpatient	79 (35)	88 (9)	
Religious Affiliation			NS
Christian	165 (73)	87 (9)	
Jewish	15 (7)	86 (9)	
Other	24 (11)	83 (16)	
None/Undesignated	22 (10)	86 (7)	

NS: Non-significant

*Possible Range: 0 (death) – 100 (perfect health)

**Figure 1:**
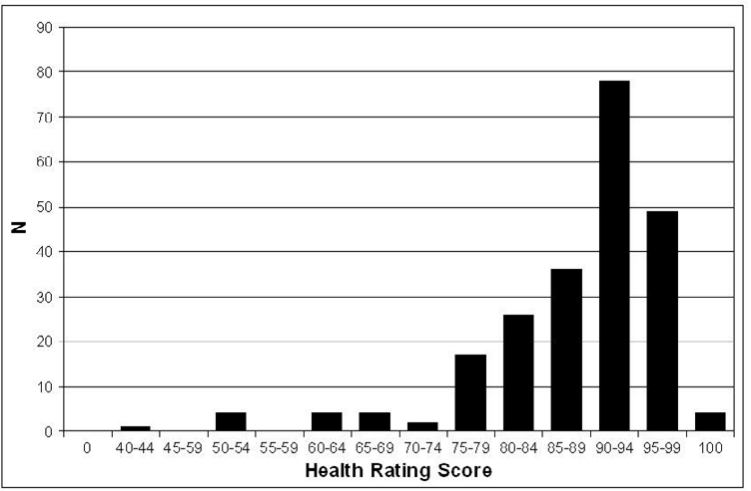
Histogram of health rating scores.

The overall mean (SD) health rating score was 87 (10; range, 40–100; Figure 1) and the median (25^th^, 75^th ^percentile) score was 90 (80, 93). Only 4 (2%) house officers rated their health as perfect. On the Likert scale, 88 (39%) stated their health was "excellent," 98 (44%) said "very good," 29 (13%) said "good," and 10 (4%) said "fair." No respondents stated that their health was "poor."

**Figure 2:**
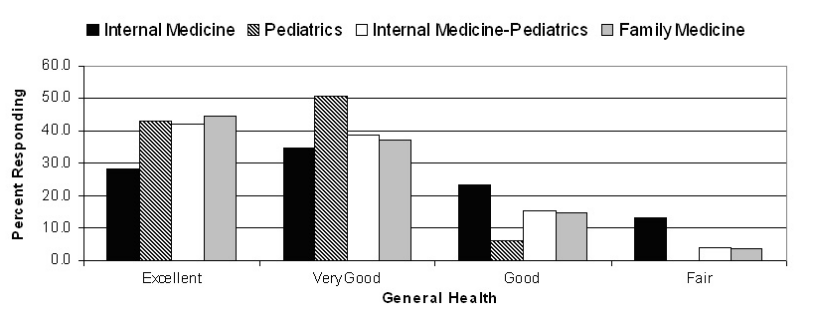
Reported general health by residency program.

In univariate analyses, residents' health rating scores were significantly related to their post-graduate year level, although there was no particular pattern. Health rating scores were also related to residency program type, with internal medicine house officers having mean scores that were 7–8 points lower than those of other house officers (Table 1). Twenty-two (37%) of internal medicine residents reported only fair or good general health (Figure 2). Greater levels of depressive symptoms (r = -0.30) and poorer spiritual well-being (r = 0.25) were associated with lower health rating scores (Table 2).

**Table 2:** Age, Health Status, Religiosity, and Spirituality: Correlations with Health Rating Scores

**Variable**	**Mean Value (SD)**	**Possible Range**	**Spearman Correlations with Health Rating Scores**	**P-value**
Age	28.7 (3.8)		0.11	NS
CESD-10	7.0 (4.7)	0 (fewest depressive symptoms) – 30 (most)	-0.30	< 0.0001
Duke Religion Index				
Organized Religious Activity	3.5 (1.3)	1 (never) – 6 (> once/week)	0.06	NS
Non-organized Religious Activity	3.0 (1.8)	1 (rarely/never) – 6 (> once/day)	0.05	NS
Intrinsic Religiosity	11.2 (3.5)	3 (low) – 15 (high)	0.05	NS
RCOPE				
Positive Religious Coping	15.1 (5.6)	6 (low) – 24 (high)	0.04	NS
Negative Religious Coping	5.2 (1.8)	4 (low) – 16 (high)	-0.08	NS
Religious Discontent	5.2 (2.3)	3 (low) – 12 (high)	-0.06	NS
Religious Support Seeking	8.3 (3.9)	5 (low) – 20 (high)	0.11	NS
Spiritual Support Seeking	11.8 (4.0)	4 (low) – 16 (high)	0.08	NS
FACIT-SpEx	71.5 (12.3)	0 (low) – 92 (high)	0.25	0.0001

NS: Non-significant; CESD-10: Center for Epidemiologic Studies-Depression scale, 10-item version;

FACIT-SpEx: Functional Assessment of Chronic Illness Therapy-Spiritual Well-being-Expanded scale

In multivariable analyses, lower health rating scores were associated with internal medicine program type, greater levels of depressive symptoms, and poorer spiritual well-being (Table 3). Residents in the internal medicine residency program reported significantly lower scores than pediatric and combined internal medicine-pediatric residents and internal medicine residents' scores trended lower when compared with those of family medicine residents (P = 0.052; parameter estimate = 4.43). The R^2 ^for the multivariable model was 0.14.

**Table 3:** Multivariable Correlates with Health Rating Scores

Significant Independent Variables*	**Parameter Estimate**	**P value**	**R**^2^
Combined internal medicine-pediatrics^†^	5.7	< 0.01	0.14
Pediatrics^†^	4.0	< 0.01	
CESD-10^‡^	-0.4	0.02	
FACIT-SpEx^§^	0.1	0.03	

CESD-10: Center for Epidemiologic Studies-Depression scale, 10-item version; FACIT-SpEx: Functional Assessment of Chronic Illness Therapy-Spiritual Well-being-Expanded scale

*Variables remained significant when the model was adjusted for age and sex

^†^Reference group: Internal medicine

^‡^Range: 0 (fewest number of depressive symptoms) – 30 (most depressive symptoms)

^§^Range: 0 (low) – 92 (high)

## Discussion

Because resident physicians are generally young and highly educated health professionals, they might be expected to report being in excellent health, especially in the context of the patients they take care of. Nevertheless, residency training is a period in which physicians deal with high levels of chronic fatigue, stress, and burnout, [18] which potentially may adversely affect the clinical care they provide.

As such, we sought to determine how residents rate their health and which factors are associated with their self-rated health. Our first finding was that self-perceived health was worse than anticipated: the mean (SD) health rating on the 0–100 scale was 87 (10), only 4 (2%) rated their health at 100, and on the Likert scale, only 39% considered their health to be excellent. In comparison, the mean (SD) health rating of adult patients with HIV was 80 (17), [36] and a representative general population sample of adults in the United Kingdom – 42% of whom reported a moderate problem in at least one dimension of their functional health – had a mean health rating of 83 (17). [37] Some previous studies had shown that women report poorer functioning than men during their training period [44] whereas other studies did not find such between-sex differences. [9,18] In our study, men and women had nearly identical health rating scores. We also found that internal medicine residents had significantly lower health ratings than pediatric and combined internal medicine-pediatric residents, and borderline-lower scores than family medicine residents (P = 0.052). On the general health question, over one-third of internal medicine residents reported that their health was only fair or good, which was about double the proportion in family medicine and internal medicine-pediatric residents, and about 6 times as great as for categorical pediatric residents (Figure 2).

Depressive symptoms were also significantly related to lower health rating scores. The correlation between psychological health and other health parameters has been described in other populations. However, depressive symptoms and other significant independent variables only accounted for a fraction of the variance in health rating scores (R^2 ^= 0.14). Other factors that we did not measure, such as subjects' personality characteristics, may have played a part in our findings. A recent review, however, found no strong associations between house officers' personality traits and risk for problems during residency training. [18] Although we wished to minimize the impact of acute sleep- and stress-related fatigue by administering the questionnaire during the In-Training Exam (no residents were on call the previous night), our findings may have been confounded by the chronic fatigue of residency. Also, we did not ask about acute illnesses (e.g., viral infections) that may have affected health ratings. Lastly, residency characteristics; lack of time for exercise, leisure activities, and health-promoting behaviors; and perceptions surrounding support from residency personnel (e.g., house staff peers, administrative personnel, faculty) may also have played a role in residents' health ratings.

We also found that certain aspects of spirituality are related to health ratings, even after controlling for mood. In multivariable analyses, those who reported poorer spiritual well-being were more likely to have poorer overall health ratings. House officers' sense of broader meaning and of connectedness may serve to buffer against the stresses and feelings of depersonalization associated with residency training, and thereby have salutary effects on perceptions of overall health. Nevertheless, religion and religiosity variables were not associated with self-rated overall health in univariate or multivariable analyses. Thus, neither the institutional/formal aspects of religion nor one's personal religious activities and religious coping mechanisms appear to influence perceptions of overall health among resident physicians. We speculate that some house officers may experience comfort and greater resilience through formal and more personal religious traditions, which may in turn positively impact their perceptions of well-being, but that time constraints and depersonalizing effects of residency training (e.g., seeing people die) may disengage others from religion. There is growing interest in teaching medical trainees [45,46] to incorporate religion and spirituality into the physician-patient relationship. However, how young physicians' own spiritual and religious needs may or may not be met in the context of their medical training has not been studied as well.

Our investigation had several limitations. The cross-sectional and non-experimental study design precludes determining causality between the significant independent factors and health rating scores. Also, we did not account for acute illnesses, such as viral infections, which may have influenced results. Lastly, although our sample was large and our response rate was high, the generalizability of our findings to other centers is uncertain.

## Conclusion

In conclusion, the self-rated health of resident physicians appears to be lower than might be expected in a presumably healthy cohort of young physicians. Self-rated health was lower for residents in internal medicine and those with a greater level of depressive symptoms and a lower level of spiritual well-being. Further work to evaluate potential resident subgroups who may benefit from interventions to improve physical, psychosocial, and spiritual/religious well-being are warranted and should also assess if such interventions have the potential to impact clinical care.

## Competing interests

Dr. Puchalski is the Founder and Director of The George Washington Institute for Spirituality and Health (GWish). GWish's stated mission is to work "toward a more compassionate system of healthcare by restoring the heart and humanity of medicine through research, education, and policy work focused on bringing increased attention to the spiritual needs of patients, families, and the healthcare professionals."

## Authors' contributions

MY, CM, SL, AP, CP, and JT participated in the conception and design of the study. MY, CM, and SL participated in the acquisition of data. MY, JM, SL, and JT participated in the analysis (see acknowledgements) and interpretation of data. MY, JM, and JT participated in the drafting of the manuscript. MY, JM, CM, SL, AP, CP, and JT participated in the critical revision of the manuscript for important intellectual content. MY, JM, SL, and JT provided administrative, technical, or material support. MY, JM, and JT provided study supervision. All authors read and approved the final manuscript.

## Pre-publication history

The pre-publication history for this paper can be accessed here:


